# Comparison of Cerebral Blood Volume and Plasma Volume in Untreated Intracranial Tumors

**DOI:** 10.1371/journal.pone.0161807

**Published:** 2016-09-01

**Authors:** Soha Bazyar, Joana Ramalho, Cihat Eldeniz, Hongyu An, Yueh Z. Lee

**Affiliations:** 1 Department of Biomedical Engineering, The University of North Carolina at Chapel Hill, Chapel Hill, North Carolina, United States of America; 2 Centro Hospitalar de Lisboa Central, Lisboa, Portugal; 3 Mallinckrodt Institute of Radiology, Washington University in St. Louis, St. Louis, Missouri, United States of America; 4 Center for Clinical Imaging Research, Washington University in St. Louis, St. Louis, Missouri, United States of America; 5 Department of Radiology, The University of North Carolina at Chapel Hill, Chapel Hill, North Carolina, United States of America; 6 Biomedical Research Imaging Center, The University of North Carolina at Chapel Hill, Chapel Hill, North Carolina, United States of America; 7 Lineberger Comprehensive Cancer Center, The University of North Carolina at Chapel Hill, Chapel Hill, North Carolina, United States of America; Henry Ford Health System, UNITED STATES

## Abstract

**Purpose:**

Plasma volume and blood volume are imaging-derived parameters that are often used to evaluation intracranial tumors. Physiologically, these parameters are directly related, but their two different methods of measurements, T1-dynamic contrast enhanced (DCE)- and T2-dynamic susceptibility contrast (DSC)-MR utilize different model assumptions and approaches. This poses the question of whether the interchangeable use of T1-DCE-MRI derived fractionated plasma volume (vp) and relative cerebral blood volume (rCBV) assessed using DSC-MRI, particularly in glioblastoma, is reliable, and if this relationship can be generalized to other types of brain tumors. Our goal was to examine the hypothetical correlation between these parameters in three most common intracranial tumor types.

**Methods:**

Twenty-four newly diagnosed, treatment naïve brain tumor patients, who had undergone DCE- and DSC-MRI, were classified in three histologically proven groups: glioblastoma (n = 7), meningioma (n = 9), and intraparenchymal metastases (n = 8). The rCBV was obtained from DSC after normalization with the normal-appearing anatomically symmetrical contralateral white matter. Correlations between these parameters were evaluated using Pearson (r), Spearman's (ρ) and Kendall’s tau-b (τ_B_) rank correlation coefficient.

**Results:**

The Pearson, Spearman and Kendall’s correlation between vp with rCBV were r = 0.193, ρ = 0.253 and τ_B_ = 0.33 (*p*-_Pearson_ = 0.326, *p-*_*Spearman*_ *= 0*.*814 and p-*_*Kendall*_ *= 0*.*823*) in glioblastoma, r = -0.007, ρ = 0.051 and τ_B_ = 0.135 (*p*-_Pearson_ = 0.970, *p-*_*Spearman*_ *= 0*.*765 and p-*_*Kendall*_ *= 0*.*358*) in meningiomas, and r = 0.289, ρ = 0.228 and τ_B_ = 0.239 (*p*-_Pearson_ = 0.109, *p-*_*Spearman*_ *= 0*.*210 and p-*_*Kendall*_ *= 0*.*095)* in metastasis.

**Conclusion:**

Results indicate that no correlation exists between vp with rCBV in glioblastomas, meningiomas and intraparenchymal metastatic lesions. Consequently, these parameters, as calculated in this study, should not be used interchangeably in either research or clinical practice.

## Introduction

Measurements of tumor hemodynamics are potentially useful for tumor characterization, as tumor aggressiveness and growth of are associated with both endothelial hyperplasia and neovascularization [1,2].

Reliable non-invasive assessment of the microvasculature and angiogenic activity can provide helpful information for diagnosis, classification, treatment planning and therapeutic monitoring of brain tumors [3–11]. Currently, relative cerebral blood volume (rCBV) assessed using dynamic susceptibility contrast- (DSC) MR imaging is the most extensively utilized perfusion measurement approach. This technique has fundamental quantification problems in lesions where there is a very leaky blood-brain barrier (BBB), namely glioblastoma multiforme (GB), and meningiomas. This error in measurement is usually corrected using additional post-processing methods, dual-echo imaging and/or a preload of contrast agent [12]. Plasma volume (vp) obtained from T1-dynamic contrast enhancement- (DCE) MR imaging is used as the surrogate of CBV when only DCE-MRI perfusion is performed. [13–15].

Physiologically, rCBV and vp should represent vascular spaces that are directly related and only differentiated by the patient’s hematocrit. To eliminate the contribution of hematocrit as a confounding factor, fractional blood volume (vb) can also be calculated. The calculations of rCBV and vp in each of these two dynamic MR imaging modalities are based on considerably different MRI contrast mechanisms. Nevertheless, both measurements reflect the distribution of contrast agent within a tissue voxel. Recently, Alcaide-Leon et al [16] reported a non-significant correlation between rCBV and vp in glioblastomas (GB). We hypothesized the T1-DCE-MRI derived vp and rCBV calculated using are DSC-MRI not correlated in brain tumor tissues, and the interchangeable use of the two parameters, especially in GB, is not reliable.

The purpose of our study was to test the hypothetical correlation between rCBV calculated from DSC and vp and vb obtained from DCE in the three most common intracranial brain tumors: glioblastomas, meningiomas, and intraparenchymal metastases.

## Materials and Methods

### Patients

The study was performed retrospectively under a University of North Carolina at Chapel Hill (UNC-CH) Institutional Review Board approved waiver. Forty-three consecutive patients were identified in our clinical Picture Archival and Storage database with histologically proven, newly diagnosed, and treatment naïve GB, meningiomas, and intraparenchymal metastatic lesions. All had undergone a standard T1-DCE- and DSC-MR imaging between October 2012 and November 2014.

From the 43 patients, nine were excluded either due to the small tumor size (n = 3), or indeterminate arterial input function (AIF) due to DSC artifacts (n = 6).

We also excluded patients who were prescribed steroids (n = 8), except those who had only received steroids on the imaging day. In the latter group, because the time of the steroid administration was not always available, we neglected the steroid effect on the BBB; similar to other published studies [16]. The final exclusion criteria were patients with significant motion between and/or during the imaging (n = 2).

Our final population (n = 24) included 18 female and 6 male (mean age 61.7±12.6 years; age range 41–89 years) with histologically proven: GB (n = 7), meningiomas (all grades) (n = 9), and intraparenchymal metastasis (n = 8). Regarding metastatic lesions, the primary tumors consisted of: breast cancer (n = 3), non-small cell lung cancer (n = 2), gastrointestinal cancer (n = 2), and ovarian cancer (n = 1). All the histopathology evaluations were performed under the supervision of experienced pathologists based on the World Health Organization criteria.

Each patient’s hematocrit value was obtained from the clinical record from a preoperative measurement within one week of the scan.

### Imaging Protocol

Imaging was performed on three identical 1.5T MR systems (Avanto, SIEMENS, Germany; Aera, SIEMENS, Germany) using 8 channel birdcage head coils. Routine pre-contrast clinical imaging was performed according to our standard protocol. The dynamic studies were acquired serially during the same imaging session without moving the patient’s head. The patients with significant head displacement between and during the imaging process were excluded. The imaging parameters were as follows: (1) pre-contrast T1-WI acquired at multiple flip angles for T1 map generation (TR = 4.1 msec, TE = 2.14 msec, FA = 2, 5, 10 and 15 degrees); (2) dynamic T1-WI (DCE) (TR = 4.1 msec TE = 2.14 msec, FA = 15 degree, 7.04 seconds per scan for 50 scans after 0.05 mmol/kg (half-dose) of Gadobenate dimeglumine (MultiHance, Bracco, Monroe Township, NJ)); (3) DSC based acquisition using a gradient echo EPI sequence (TR = 2sec, TE = 54 msec, FA = 60 degrees, 60 measures) after a second half-dose of 0.05 mmol/kg of Gadobenate dimeglumine. In both dynamic evaluations, intravenous gadolinium-based contrast agent was injected after the completion of the fifth repetition. A standard high-resolution post-contrast T1-weighted (MP-RAGE) was performed afterward as part of the standard protocol. Contrast agent was administered intravenously using an automated power injector at 5 mL/s, followed by 18 mL saline bolus injected at 5 mL/s. Imaging data was then transferred off-line for analysis.

### Image Analysis

**Fig 1:**
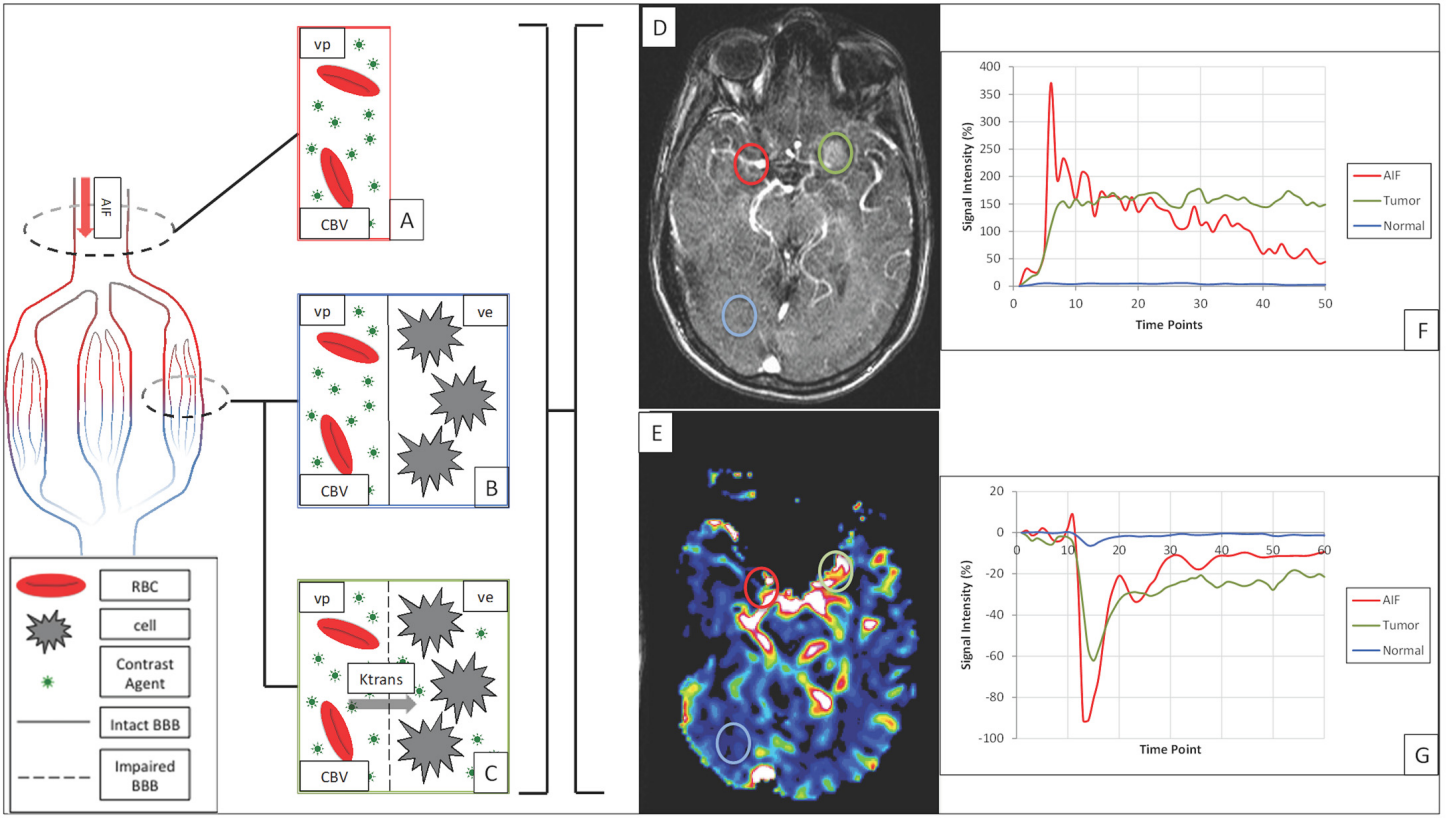
Schematic picture of the mechanism behind contrast signal changes during dynamic MR imaging. D and E are axial DCE- and DSC-MR images of a meningioma patient, respectively. Graphs (F,G) demonstrate the signal intensity (SI) (%) at different time points. Gadolinium-based contrast agents increase the T1 (F) and reduce T2* (G) SI. The change in SI is generally related to blood concentration. The SI in AIF region (D,E: red circles) changes rapidly and dramatically (F,G: red line) as the bolus dose of contrast agent reaches the brain vessels in the first path after injection and wash out fast (F,G: red line, first spike). In tumor tissue (D,E: green circles), through leaky BBB (C), contrast agent enters (F,G: green line, steep slope section) and entraps (F,G: green line plateau section) in the interstitial space (ve), while in normal brain tissue (D, E: blue circles), due to intact BBB (B) SI changes are minimal (F,G: blue line).

Automated post-processing of T1-DCE data was performed on a Versavue workstation with Omnilook^®^ (iCad, Nashua NH), based on Tofts three-parameter fit model [17,18] and Weimann [19] population-based arterial input function (AIF). The Weimann AIF was measured from venous blood starting at 1min post contrast injection, at 2min, and exponentially increasing intervals in healthy volunteers [19]. The main advantage of population-based AIF approach is that it allows for quantitative assessment of tissue properties without the need to obtain high temporal resolution images to characterize an AIF. This results in acquiring images with higher spatial resolution and/or SNR, and increasing the ability to probe tissue heterogeneity [20]. The T2*-based-DSC data was processed using an ImageJ plug-in developed in-house. First pass concentration-time curves were computed voxel-by-voxel using the DSC images. Using the concentration-time curve, a voxel was manually selected in an appropriate slice adjacent to the contralateral middle cerebral artery as AIF (Fig 1E and 1G). An integration of the first pass concentration-time curve was then performed for each voxel and normalized by the AIF curve to obtain CBV. The perfusion images were then transferred to the Versavue^®^ workstation for subsequent analysis.

ROIs were manually delineated along the enhancing margins of lesions on T1-DCE-MR images by one reader (S.B.) under the supervision of an expert neuroradiologist (Y.Z.L.), both blinded to histology and clinical data (Fig 1D). The shape of the ROI was guided by the lesion shape and ranged between 30–40 pixels. Four regions of interest were identified per lesion to sample various portions of the lesion with the highest enhancement. Macroscopic vessels, necrotic and hemorrhagic areas (if present and as defined on conventional imaging) were avoided. In the case of multiple metastatic lesions, the largest lesion was chosen to draw the ROIs.

The mean vp was measured in each ROI. Mean volume transfer coefficient (Ktrans) (min^-1^) was also measured because it might have an influence on vp calculation and consequently, vb values [21]. Since both dynamic series were acquired in the same session without moving the patient’s head, the same ROI was propagated to DSC-MR images to evaluate the anatomically identical corresponding CBV (Fig 1E). The rCBV was calculated based on the mean CBV extracted from an ROI that was manually drawn on the symmetrical, normal-appearing contralateral white matter (Fig 1E). This normalization made the rCBV independent of individually different AIF. vb (%) was calculated using the following equation:

$$vb=\frac{vp}{\left(1-HCT\right)}$$

where vp(%) and HCT represents plasma volume and patient hematocrit, respectively.

### Statistical Analysis

Statistical analysis was performed by IBM SPSS version 22 (IBM, Armonk, New York). A p-value < 0.05 was considered statistically significant. To test the normal distribution, the Shapiro–Wilk test was performed. Non-parametric tests, Kruskal–Wallis one-way analysis of variance and chi-square, were used to assess differences in numerical and nominal variables within the different groups, respectively. Pearson (r) and Spearman’s rank (ρ) and Kendall’s tau-b rank (τ_B_) correlation coefficient were performed to evaluate the relationship between vp and vb with rCBV within all groups. The Pearson correlation evaluates the linear relationship between two continuous variables. Both Spearman and Kendall’s tau are non-parametric tests that evaluate the monotonic relationship. Although the Spearman approach is the most widely accepted and utilized method, for small sample sizes, Kendall’s tau is less prone to errors. When both are computed, if ρ or τ_B_ > r, the correlation is monotonic but not linear. The strength of the correlation was considered as perfect if r, ρ, τ_B_ = 1; strong if 0.7 ≤ (r, ρ, τ_B_) < 0.1; moderate if 0.5 ≤ (r, ρ, τ_B_) < 0.7; weak if 0.3 ≤ (r, ρ, τ_B_) < 0.5; and no relationship if (r, ρ, τ_B_) ≤ 0.3, as our aim was to evaluate the positive correlation between vp and rCBV.

## Results

The demographics of our study population are shown in Table 1. There were no significant differences in gender and age between groups. The significant difference in hematocrit could be anticipated due to the physiological difference in gender distribution among the groups (Table 1).

**Table 1:** Demographics of study population in the three diagnostic groups.

Diagnosis	Glioblastoma	Meningioma	Metastasis	p-value
**Age (year)**	67.86±14.47	54.44±9.82	64.38±11.03	0.073
**Gender**	4 F+3 M	8 F+ 1 M	6 F+ 2 M	0.347
**Hematocrit (%)**	40.16±3.24	34.53±8.27	34.63±3.84	0.048*

*p-value < 0.05

**Fig 2:**
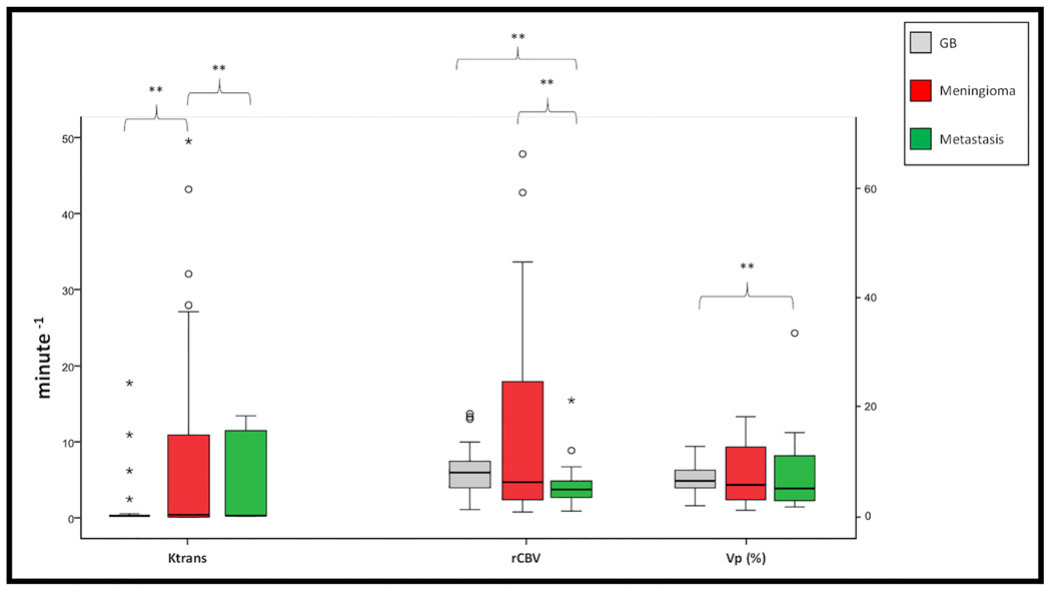
Box-and-Whisker plot of permeability and perfusion parameters. The graph demonstrates median, maximum, minimum and first and third quartile of Ktrans (min^-1^), relative cerebral blood volume (rCBV) and fractional plasma volume (VP) (%) in three different types of brain tumor. (**p-value<0.05)

A total of 96 ROIs were designated along the enhancing margins of lesions 24 enhancing lesions (four ROIs on each lesion). T1-weighted leakage (rising the post-contrast T2/T2*-signal above the pre-contrast baseline) was not detectable, although post-contrast signal remained below its pre-contrast baseline in DSC signal intensity curves (T2/T2* weighted residual effect) (Fig 2G).

**Fig 3:**
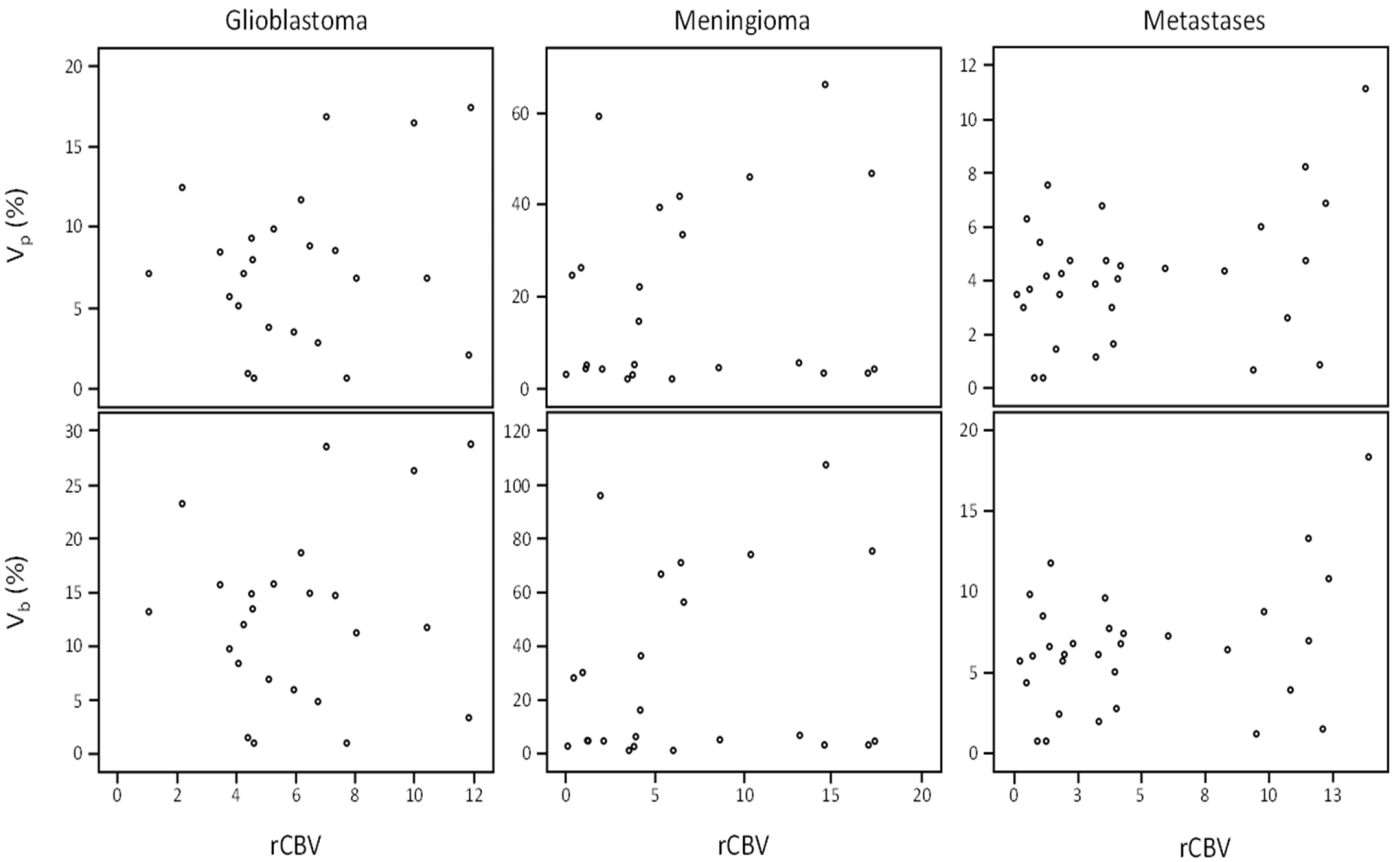
Graphical relationship between the perfusion parameters in different study groups. Scatter-plot of fractional plasma volume (vp) (%), fractional blood volume (vb) (%) and relative cerebral blood volume (rCBV) in glioblastoma, meningioma, and intraparenchymal metastatic lesions.

The Shapiro–Wilk test demonstrated that the perfusion parameters and Ktrans distributions were non-Gaussian (data not shown). Median and interquartile range of rCBV, vp, and Ktrans are shown in Fig 2. Interestingly, both rCBV and vp were significantly higher in GB than metastatic lesions (p_rCBV_ = 0.031 and p_vp_ = 0.001, respectively) (Fig 2). Ktrans in meningiomas (median_ktrans_ = 0.36) was found to be significantly higher than glioblastomas (median_ktrans_ = 0.12) (p_ktrans_ = 0.000) and metastasis (median_ktrans_ = 0.09) (p_ktrans_ = 0.04). The relationship between vp or vb with rCBV were evaluated using Pearson, Spearman and Kendall’s tau correlation and the correlation coefficients and p-values are listed in Table 2. The results showed that both vp and vb were not significantly correlated with rCBV in GB, meningiomas, and metastasis, regardless of hematocrit and blood volume compensation (Fig 3).

**Table 2:** Analyze of correlation between perfusion parameters.

	vp (%) vs. rCBV						vb (%) vs. rCBV					
	Pearson		Spearman		Kendall		Pearson		Spearman		Kendall	
	r	p-value	ρ	p-value	τB	p-value	r	p-value	ρ	p-value	τB	p-value
**GB**	.193	.326	.253	.814	.033	.823	.160	.416	.043	.828	.018	.901
**Mng**	-.007	.970	.051	.765	.135	.358	.023	.895	.060	.728	.145	.321
**IPM**	.289	.109	.228	.210	.239	.095	.094	.301	.251	.166	.234	.112

fractional blood volume, vb; rational cerebral blood volume, rCBV; fractional plasma volume, vp; Pearson correlation coefficients, r; Spearman’s rank correlation coefficients, ρ; Kendall’s tau-b rank correlation coefficient, τB; Glioblastoma multiformis, GB; Meningioma, Mng; Intraparenchymal Metastases, IPM.

## Discussion

The goal of our study was to explore the correlation between rCBV and vp, derived from DSC- and DCE-MRI approaches, in three most common types of intracranial tumors. Our results showed that rCBV derived from DSC- and vp obtained from DCE-MRI were not correlated in glioblastomas, meningiomas, and intraparenchymal metastatic lesions (Table 2). The lack of correlation persisted even after correction for the vascular space differences caused by hematocrit. This raises the concern that though the two parameters are physiologically related, the calculated values using above-mentioned dynamic MR-imaging modalities may measure different vascular processes and cannot be used interchangeably.

When compared with previously published our results were in reasonable agreement [10,16,21–25]. For example, the mean rCBV value in our study for glioblastoma and metastasis obtained with the DSC-MRI method were similar to the mean rCBV reported by Servers et al [21]. We also found very similar vb for metastasis as reported by Lüdemann et al [10]. Santarosa et al [23] reported the median value of the vp in high-grade glioma similar to this study. In addition, the median and interquartile range of Ktrans in our study groups were also congruent with previously published studies [16,24,25]. The pharmacokinetic model of Tofts and Kermode fits a triexponential enhancement curve to a theoretic model based on compartmental analysis after a standard dose of gadolinium-based contrast agent. This model does not necessarily apply in highly vascular extra-axial tumors and complicates the calculation of Ktrans [26]. This may explain the wide range of values for Ktrans (0.51–49.49) in meningioma in the current study (Fig 2).

The measured mean values of vp, vb and rCBV in our study were higher in glioblastomas than metastatic lesions (Fig 2). Several other studies also found a higher rCBV in glioblastomas compared to metastases [21,22,27]. There are several reasons for increased blood volume in glioblastomas and metastases. Glioblastomas and metastases are very heterogeneous and the angiogenesis process is very complex, with the potential for “failure” as evidenced by tissue necrosis in treatment naïve tumors. As shown by molecular stains, such as CD34, there is an increase in the number of tortuous vessels, giving rise to a ‘‘swamp” of numerous, small slow-flowing vessels in gliomas [28]. This factor is important, as antiangiogenic therapy has been shown to ‘‘normalize” these vessels, increasing the diameter and thereby increasing cerebral blood flow (CBF) and decreasing CBV (due to a decrease in overall vessel density) [15]. This vessel “swamp” may also play a role in the higher values of vp and rCBV in glioblastomas compared to metastases (Fig 2). On the other hand, new vessel growth in brain metastases is likely related to the biology of the primary tumor types, a hypothesis that may be explored in future studies. Multiple studies have attempted to differentiate glioblastomas from metastases using perfusion and permeability measurements derived from DSC and DCE-MRI [10,21,22]. Server et al [21] did not find statistically significant differences when comparing tumor rCBV in glioblastomas vs. metastases. We observed that the rCBV (p = 0.031) and vp (p = 0.001) were capable of differentiating these two lesions, but not the Ktrans (p = 0.375), since glioblastomas posed significantly higher perfusion (Fig 2). Previous studies showed similar findings regarding Ktrans [10] and perfusion [29]. However, these results are controversial. The physiological mechanism behind them is not fully understood and is beyond the scope of the current study.

As stated before, the vp and the CBV are related via vb, and the hematocrit in the vb calculation reflects the hematocrit of the microvasculature. Based on Fahraeus effect [30], the value of hematocrit is lower in small vessels compared to large ones, which may affect the Ktrans, vp and rCBV measurements. However, this effect has been considered negligible by some [31]. One would anticipate that the rCBV and vb are equal or at least directly related when measured in the same ROIs. However, we found no correlation between either vp or vb with rCBV in glioblastomas, meningiomas and metastatic lesions (Table 2, Fig 3) and our data suggests that the two parameters may provide different physiological information.

There are different hypothetical reasons for inequality between vp and vb with rCBV. First, an accurate measurement of rCBV, using the standard DSC modeling approach, relies on intravascular localization of contrast agent, which is compromised by the leaky BBB of brain tumors (Fig 1B and 1C). Gadolinium contrast agents reduce T2* signal intensity during a dynamic first-pass injection and it is also an effective T1 relaxation enhancer (Fig 1F and 1G). Extravasation of contrast agent into the extravascular/extracellular space (ve) in enhancing tumors can confound the rCBV estimation obtained using DSC-MRI [32]. Therefore, DSC-MRI using gadolinium can falsely estimate low rCBV in some patients. Preloading with a small dose of contrast is an empirical correction method that minimizes the effect of T1-weighted leakage [33–35]. The preload dose administration before DSC leads to saturation of ve, and consequently decreases the intensity of T1-induced signal during the subsequent DSC contrast injection [34,35]. Paulson et al [33] reported the preloading as one of the robust methods to overcome the “leakage effect”. Aside from dose, the incubation time that allows the preload contrast agent to diffuse into ve also plays an important role in adequate correction [33]. Immediate imaging after the preload administration even by a maximum dose of the contrast would not have the sufficient effect on T1 leakage effect [33, 35, 36]. In the current study, a half dose of gadolinium was injected for acquiring T1-DCE-MR imaging; which also served as a preloading dose for the second half contrast injection whereby the DSC-MR images were acquired. Using this method, no T1-weighted leakage was detected in DSC-MR imaging signal (Fig 1G).

Secondly, amplification of the intravascular T1 signal from the extravasated contrast agent, may overestimate vp in DCE-MRI [2,9,37]. It has been suggested that the analysis of DCE-MRI dataset with the commonly accepted pharmacokinetic model of Tofts and Kermode [17], led to the systematic overestimation of Ktrans and potentially large underestimation of the vp [38, 39]. Different approaches for the estimation of permeability parameters derived from DCE-MRI leads to different results [31]. Harrer et al [40] applied three kinetic models to estimate Ktrans and vp derived from DCE-MRI and concluded that the methods that incorporate a measured AIF and an estimate of vp allow for a more accurate estimation of permeability. Thus, in the current study, the most commonly available and FDA approved approach was utilized, and it was beyond the scope of our study to explore the correlations with other post-processing techniques.

Alcaide-Leon et al [16], in a study on glioblastoma, found a weak correlation (ρ = 0.382, p<0.001) between vp and rCBV in a pixel-by-pixel comparison in the entire enhancing region of the tumor. This low correlation was likely accentuated by either spatial misregistration or hemosiderin deposition. Using the pixel-wise comparison, rendered their approach susceptible to noise and signal fluctuation, even though they discarded the outlier data before analysis. Furthermore, that study did not include the pre-contrast T1 sequences, and used a fixed baseline T1 value to calculate Ktrans. As mentioned before, accurate Ktrans evaluation is needed because it may have an influence on vp calculation and consequently, vb values [21]. Haroon et al [41] also compared the T2*-DSC and T1-DCE CBV and found a moderate correlation between these two techniques (ρ = 0.667, p<0.05), but their study group was very small, and different grades of glioma and other types of intra-axial tumors were not compared separately. In addition, their ROIs were placed on the entire enhanced part of the lesion and a different algorithm was used to analyze their images. Lüdemann et al [2] also found a very weak insignificant correlation (r = 0.407, p = 0.060) in a pixel-by-pixel comparison between tumor perfusion calculated using the aforementioned dynamic MR imaging modalities. To the best of our knowledge, this is the first study that systematically compares vp determined by DCE-MRI with rCBV extracted from DSC in different brain tumor types. Prospective validation of these preliminary results is required to confidently establish the differences between rCBV and vp measurements, since they are not correlated with each other as strongly as expect. Additional work is needed to determine tumor vascular parameters correctly and uniformly and develop a consensus for the methodology amongst various institutions.

Aside from the retrospective designed and small sample size, another limitation of our study was the use of corticosteroids. We excluded patients who were on steroids and only retained patients who received steroids on the imaging day. The exact time of steroid administration was not available, so similar to other authors [16], we assumed that the steroids effect was negligible for this small number of patients (n = 2). We used a general concentration-time curve (also used by Tofts and Kermode [17] and originally described by Weinmann et al [19]) instead of individuals AIF for DCE-MR image data processing. The main advantage of this approach is that it allows for quantitative assessment of tissue properties without the need to obtain high temporal resolution images to characterize an AIF, making it a more clinically relevant approach. This permits acquiring images with higher spatial resolution and/or SNR [20] due to the reduced coverage needed to obtain an AIF. Bergamino et al [42] found no significant differences in Ktrans and vp value processed using Tofts and Kermode model either by using standard AIF or individual selected AIF. While Li et al also used this method for processing the DCE-MR images [43], so we assumed this error is negligible. The algorithm to correct rCBV in regard to T2/T2* weighted residual effect was not used in this study. Though recent publications have demonstrated that CBV measurement accuracy increases by utilizing such algorithm, [34,35] our goal was to compare the DCE and DSC perfusion parameters used in a clinical setting, so we used the conventional method.

In conclusion, our results demonstrated that rCBV and vp values, derived from DSC and DCE, respectively, as calculated in our study, were not correlated. This finding emphasizes that these two measurements cannot be used interchangeably in clinical practice. Further work is needed to compare DCE- and DSC-MRI perfusion data with actual physiological values for true validation, but until then, an imaging protocol combining both of these measurements may provide a more thorough evaluation of intracranial tumor perfusion.
